# Associations of coping and health-related behaviors with medical students’ well-being and performance during objective structured clinical examination

**DOI:** 10.1038/s41598-024-61800-1

**Published:** 2024-05-17

**Authors:** Noémie Barret, Théodore Guillaumée, Thomas Rimmelé, Marion Cortet, Stéphanie Mazza, Antoine Duclos, Gilles Rode, Marc Lilot, Sophie Schlatter

**Affiliations:** 1 Lyon Est School of Medicine, Université Lyon 1, Lyon, France; 2 Department of Anesthesia and Intensive Care, Edouard Herriot Hospital, Hospices Civils de Lyon, Lyon, France; 3 High Fidelity Medical Simulation Center (SIMULYON), Lyon, France; 4 Department of Obstetrics and Gynecology, Croix Rousse Hospital, Hospices Civils de Lyon, Lyon, France; 5 Centre de Recherche en Neurosciences de Lyon (CRNL), CRNL-U1028, UMR-5292, FORGETTING Team, CNRS, INSERM, Université Lyon 1, 69500 Bron, France; 6 Research on Healthcare Performance RESHAPE, INSERM U1290, Université Claude Bernard, Lyon 1, France; 7 Department of Health Data, Hospices Civils de Lyon, Lyon, France; 8 Université Claude Bernard Lyon 1, CNRS, INSERM, Centre de Recherche en Neurosciences de Lyon CRNL U1028 UMR5292, TRAJECTOIRES, F-69500 Bron, France; 9 Unit of Pediatric and Congenital Cardio-thoracic Anesthesia and Intensive Care, Louis Pradel Hospital, Hospices Civils de Lyon, Lyon, France

**Keywords:** Academic, Avoidance, Cope, Medical students, Positive thinking, Performance, Physical activity, Sleep, Stress, Health care, Human behaviour, Emotion, Stress and resilience

## Abstract

Objective structured clinical examination (OSCE) is a valid method to evaluate medical students’ competencies. The present cross-sectional study aimed at determining how students’ coping and health-related behaviors are associated with their psychological well-being and performance on the day of the OSCE. Fourth-year medical students answered a set of standardized questionnaires assessing their coping (BCI) and health-related behaviors before the examination (sleep PSQI, physical activity GPAQ). Immediately before the OSCE, they reported their level of instant psychological well-being on multi-dimensional visual analogue scales. OSCE performance was assessed by examiners blinded to the study. Associations were explored using multivariable linear regression models. A total of 482 students were included. Instant psychological well-being was positively associated with the level of positive thinking and of physical activity. It was negatively associated with the level of avoidance and of sleep disturbance. Furthermore, performance was negatively associated with the level of avoidance. Positive thinking, good sleep quality, and higher level of physical activity were all associated with improved well-being before the OSCE. Conversely, avoidance coping behaviors seem to be detrimental to both well-being and OSCE performance. The recommendation is to pay special attention to students who engage in avoidance and to consider implementing stress management programs.

*Clinical trial*: The study protocol was registered on clinicaltrial.gov NCT05393206, date of registration: 11 June 2022.

## Introduction

Medical students face numerous stressful situations, such as night shifts, proximity to death, and hyper competitive examinations^1–3^. The exposure to those stressors might lead to intense responses represented by emotional, physiological, and cognitive changes that affect short-term well-being and performance, as well as long-term health^4,5^. However, students differ in their level of stress vulnerability, and the intensity of their emotional response might be dependent of their conscious and/or unconscious behaviors (e.g., coping and health-related behaviors)^6,7^.

Coping has been defined as “cognitive and behavioral efforts to manage specific external and/or internal demands that are appraised as taxing or exceeding the resources of the person”^6^. Four main categories of coping behaviors have been described: social support, positive thinking, active resolution, and avoidance^7^. Engaging in such coping behaviors can modify individuals’ levels of inner resources and influence their stress responses. For example, social support and positive reinterpretation are known to be protective factors against stress and anxiety^8^; conversely, avoidance, which is described as maladaptive coping behaviors, increases stress levels^6,9^. Further findings also suggest that coping behaviors may influence performance^10^. For instance, it has been shown that problem-solving (which is part of the active resolution) could be associated with better academic achievement, while avoidance was associated with decreased performance in medical students^11^.

Like coping behaviors, health-related behaviors can also modify the levels of inner resources available to cope with a stressful situation^12^. Among them, sleep quality and physical activity have a considerable influence on stress responses^13–15^. In medical students, poor sleep quality and insomnia have been associated with a high stress level and poor academic performance^16–18^. A lack of physical activity has also been found to worsen stress levels^19^, and to be one of the most predictive factors of burn-out symptoms in medical students^20^.

Among the stressful factors faced by medical students, ranking examinations are known to be one of the most anxiety-provoking events^21^. Objective structured clinical examination (OSCE), which is based on clinical simulation, is a valid method for the evaluation of medical skills^22,23^. It will soon play a major role in the evaluation of French medical students as its results will weigh heavily on their national ranking, which then determines the medical specialty of their fellowship. Traditional exams typically assess students' knowledge outside the context of clinical practice, with grading occurring after the exams and without interaction between students and assessor. In contrast, the OSCE is a clinical examination where students demonstrate specific professional skills under direct evaluation. As a result, it is expected that the OSCE would induce a stress due to the direct judgments of observing assessors, in addition to the stress associated with any exam.

Due to the specific requirements of this exam, stress management is expected to be considered important, even crucial, for students' future success and is important to understand how students cope with the stress that is generated. Two recent studies explored the relationships between coping, stress, and performance in a context of medical training in simulation^24,25^. These studies offer interesting findings, yet they focused on small cohorts of medical students participating in high fidelity simulation sessions without academic expectations. Thus, associations between coping strategies and stress in mandatory OSCE remain to be explored.

Moreover, investigating the relationships between psychological well-being, performance, coping, and health-related behaviors in this context would enable to pave the way for recommendations and programs designed specifically for medical students. Well-being remains a complex concept without a consensual definition^26–28^, Burns states that “psychological well-being refers to inter- and intra-individual levels of positive functioning that can include one’s sense of mastery and personal growth”^29^. In the present study, we use the term instant psychological well-being to characterize a state of low level of stress, with a stress perceived positively, and high self-confidence.

The purpose of this exploratory study was to describe how medical students’ coping and health-related behaviors were associated with their instant psychological well-being on the day of the OSCE. The secondary aim was to describe how medical students’ coping and health-related behaviors were associated with OSCE performance. All research questions were exploratory in nature.

## Methods

### Study design and population

This cross-sectional, observational study involved all fourth-year medical undergraduate students who participated in mandatory OSCE scheduled at the Lyon Est school of Medicine, from the 15th to the 17th of May 2022. All students signed a written consent form before inclusion. There were no exclusion criteria.

### Ethical aspects

This study is part of a larger project called ECOSTRESS. This research project was co-constructed by the health services of the Lyon 1 University, the local medical students' association, and the dean of the Lyon Est school of Medicine. All students were informed about the course of the study and its overall aim (i.e., determine factors that influence well-being and performance in order to develop appropriate tools for medical students in the future). Six investigators provided information about the study, collected signed consent forms, and enrolled students. No monetary compensation was provided. The study was conducted in accordance with the Declaration of Helsinki and was approved by the Institutional Review Board of the Lyon 1 University (France, IRB 2020-05-12-01). The study protocol was registered on clinicaltrials.gov before any inclusions (clinical trial ID: NCT05393206).

### Timeline

The experiment spanned 3 days. Over these 3 days, 483 students came to their exam. Before and after their exam, students who voluntarily took part in the experiment (n = 482) answered a survey split into 2 parts: some questionnaires were answered just before the exam (5 min) and others following the exam (20 min). Students came in groups of 20, therefore 20 students answered the survey simultaneously. Each student was seated at an individual desk with the computerized questionnaire on a laptop. The students were arranged to ensure that their neighbors did not see their answers.

Just before starting the OSCE, students answered several visual analogue scales (VAS) informing on their instant psychological well-being. After the OSCE, students answered a set of questionnaires to report how they behaved in terms of coping, sleep, and physical activity before their examination. Demographic information (age, gender, height, weight) was also collected.

### OSCE

During OSCE, students are convened to demonstrate, with a time constraint of a few minutes, several medical competencies during consecutive realistic scenarios involving standardized patients and/or manikins. The competence evaluated through OSCE includes several clinical abilities such as practical knowledge, skills (including communication), and demonstration of professional attitudes. The national ranking exam scheduled for all sixth-year medical students in France in 2024 will incorporate an OSCE, which will contribute 30% to the total score, affecting students' national ranking and their selection of medical residencies. This local OSCE served as a summative exam, constituting 20% of the evaluation of medical students' clinical abilities during their clerkship. It was conducted under similar organizational conditions to those of following national ranking OSCE (Appendix 1).

### Assessment of indicators of instant psychological well-being on the day of the OSCE

Just before their examination, students reported their instant psychological well-being on three VAS of 100 mm (Appendix 2)^30–32^. First, they reported their stress level on a scale from 0 “zero stress”, to 100 “maximum stress” (stress). Second, they reported the emotional valence of their stress, which is the way they perceived the stress, from 0 “the most negative feeling possible” (distress), to 100 a state associated with “the most positive feeling possible” (eustress) (emotional valence). Third, they reported their level of self-confidence relative to the upcoming examination, from 0 “zero self-confidence” to 100 “maximum self-confidence” (self-confidence). From these three variables, a score of instant psychological well-being was calculated as follows:

$${\text{Instant psychological well-being}} = \frac{{\left( {100 - {\text{stress}}} \right) + {\text{emotional valence}} + {\text{self confidence}}}}{3}$$

The score may range from 0 to 100; a high score indicates a high level of instant psychological well-being.

### Assessment of coping behaviors

The brief cope inventory (BCI) is a 28-item questionnaire that was used to identify the coping behaviors of the students during the 2 weeks prior to the OSCE^33^. Answers are given using a 4-point Likert scale. Four strategies of coping behavior are extracted from the answers: active resolution (e.g., planning, active coping), positive thinking (e.g., humor, acceptance, positive reframing), social support (e.g., emotional support, venting, religion), and avoidance (e.g., denial, behavioral disengagement, substance use)^7,33^. Each strategy of coping behavior was scored from 1 to 4, a high score indicating the behavior was highly engaged. Each BCI scores for may range from 1 to 4.

### Assessment of health-related behaviors

The Pittsburgh sleep quality index (PSQI) is a 19-item questionnaire used to identify sleep disturbance^34,35^. Students were asked about their sleeping habits during the month preceding the OSCE. A high score indicates elevated sleep disturbance; a score ≥ 6/21 was considered as the sleep disturbance threshold^36^. The score may range from 0 to 21.

The global physical activity questionnaire (GPAQ), developed by the World Health Organization (WHO), is composed of 6 main questions^37^. The GPAQ identifies the physical activity and sedentary behaviors (defined by the number of hours spent sitting or reclining on a typical day, not including time spent sleeping) during a regular week. The metabolic equivalent of task (MET) is the ratio of the metabolic rate of a person doing physical activity, relative to their resting metabolic rate (during quiet sitting)^37^. The MET is used to express the intensity of physical activity (e.g., moderate activity corresponds to 4 MET). The sum of all activities is given in MET-minutes per week. The lowest score is 0 (i.e., no activity at all) and there is no upper limit. The WHO recommendation on physical activity for health is 600 MET-minutes per week, which corresponds to 150 min of moderate intensity physical activity^38^. Questionnaires with missing data and/or aberrant responses were excluded from further analysis^38^.

### Assessment of performance in the OSCE

Each OSCE was composed of 5 different scenarios and lasted 50 min. Scenarios covered a wide range of medical practices and used standardized patients or specific manikins/phantoms for procedural techniques (surgical suture, insertion of intravenous line, otoscopy) (Appendix 1). OSCE performance was assessed through standard grids by university examiners independent of the research project. The total OSCE score of each student (ranging from 0 to 40 points) was the mean of the scores of the 5 scenarios.

### Statistics

Two different multivariable linear regression models were constructed. The first explored the relationships between instant psychological well-being with coping behaviors (active resolution, positive thinking, social support, avoidance) and health-related behaviors (sleep disturbance, physical activity). The second explored the relationships between academic performance (OSCE score) with coping and health-related behaviors. Both models were controlled for gender and age. As there is extensive literature linking higher BMI to lower subjective well-being^39,40^, the models additionally controlled for BMI. The normality of data distribution was explored using histograms and quantile–quantile plots and models’ assumptions were checked (Appendix 3). The β coefficients with their 95% confidence interval (i.e., the degree of change in the outcome variable for every 1 unit of change in the predictor variable) and the adjusted coefficients R^2^ (i.e., percentage of variance explained) are provided for all regression models. Additional effect sizes, along with their 95% CI, have been calculated for individuals’ predictors using the effect size package (v0.8.6). For further analysis, correlations between all coping and health-related behaviors and the three sub-components of the instant psychological well-being (stress, emotional valence, self-confidence levels) and performance were performed. For ethical reasons, participation to the protocol was offered to all the students that were scheduled to the OSCE (n = 493), no other a priori sample size was calculated. Data were analyzed using the R software (v4.1.2). All hypotheses were tested using a statistical significance level of 0.05. Quantitative data are presented by mean (standard deviation, SD) or median [interquartile range, IQR] according to the distribution of the data, and nominal data by n and percentage.

### Ethics declarations

The research was performed in accordance with the Declaration of Helsinki and has been approved by an appropriate ethics committee, that is the Institutional Review Board of the Claude Bernard University Lyon 1, Lyon, France (no. IRB 2020_05_12_01, December 2020) and written informed consent was obtained from all participants.

## Results

### Students’ characteristics

**Figure 1:**
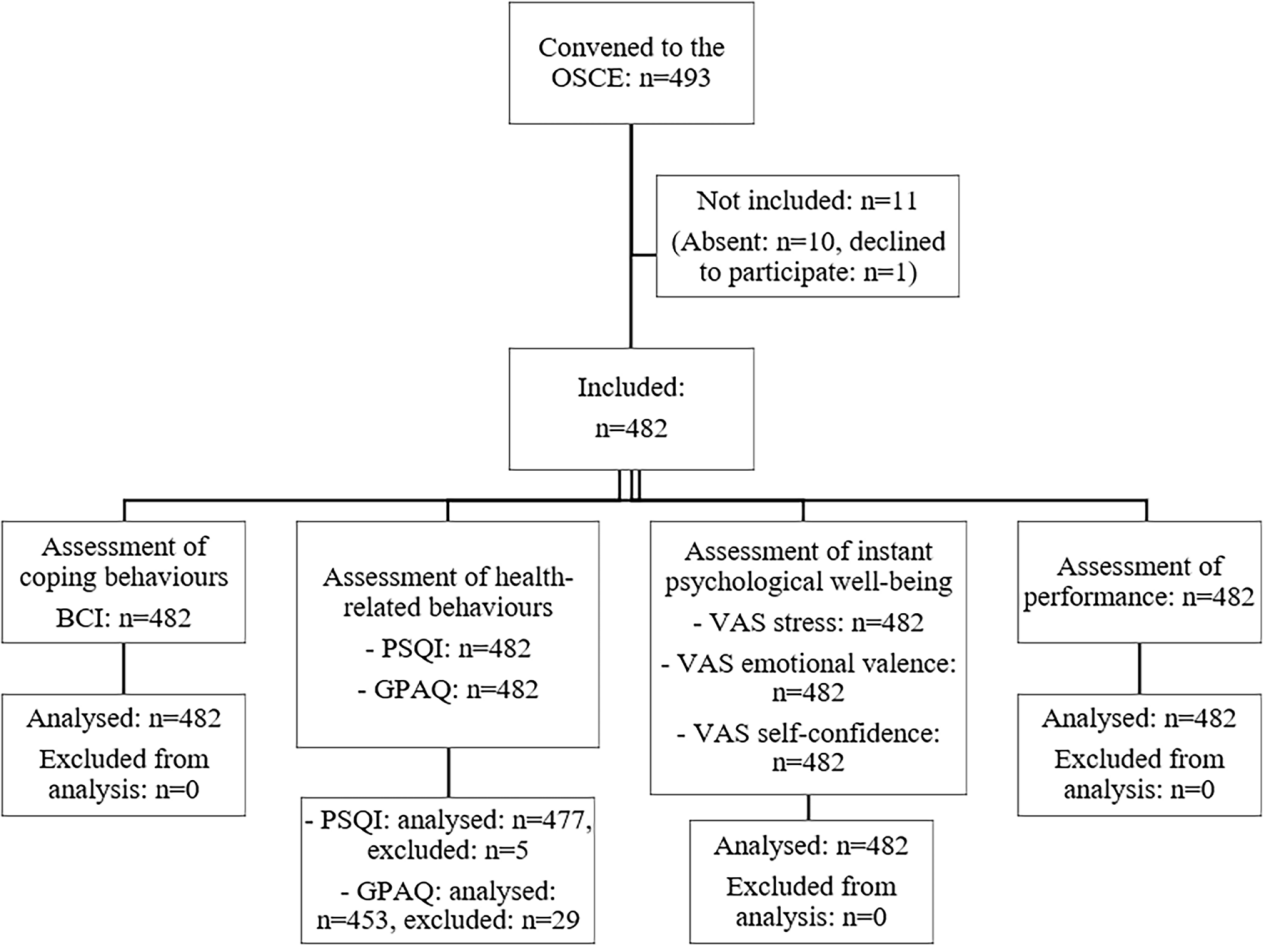
Flow-chart of the studied population: all 4th-year medical students convened to the academic objective structured clinical examination (OSCE). *BCI* brief cope inventory, *PSQI* Pittsburgh sleep quality index, *GPAQ* global physical activity questionnaire, *VAS* visual analogue scale (100 mm). Some PSQI (n = 5) and GPAQ (n = 29) student questionnaires were excluded from further analysis due to aberrant answers.

A total of 493 students were convened to the OSCE, 10 of whom did not attend the examination and 1 declined to participate; 482 were included in the present study (Fig. 1). Cronbach’s alpha values were all over 0.70 (0.86 for active resolution, 0.71 for positive thinking, 0.82 for social support, and 0.76 for avoidance), indicating a good internal consistency. The most executed coping behavior was positive thinking (M ± SD; 2.64 ± 0.64), followed by active resolution (2.07 ± 0.74), social support (1.97 ± 0.59), and avoidance (1.59 ± 0.42; Table 1). Regarding health-related behaviors, 93% of students had a sufficient level of physical activity and 53% reported sleep disturbance (Table 1). The detailed results of the PSQI and GPAQ questionnaires are presented in Appendices 4 and 5.

**Table 1:** Demographic and descriptive characteristics of the students (n = 482).

Demographic characteristics	
Gender	
Female, n (%)	314 (65)
Male, n (%)	166 (34)
Other, n (%)	2 (0.4)
Age (years), Mdn [IQR]	22 [21, 23]
Body mass index (kg/m^2^), Mdn [IQR]	21.51 [19.89, 23.38]
Coping behaviors	
Active resolution, Mdn [IQR]	2.0 [1.5, 2.5]
Positive thinking, Mdn [IQR]	2.7 [2.2, 3.2]
Social support, Mdn [IQR]	1.9 [1.5, 2.4]
Avoidance, Mdn [IQR]	1.9 [1.5, 2.4]
Health-related behaviors	
Physical activity level (MET-minutes/week), Mdn [IQR]	2400 [1260, 3720]
Meet the WHO threshold for minimal physical activity, n (%)	421 (93)
Sedentary behaviors (hours/day), M (± SD)	10.5 (± 3.5)
Sleep disturbance level, Mdn [IQR]	6.0 [4.0, 8.0]
Positive sleep disturbance screening, n (%)	252 (53)
Smoker, n (%)	77 (16)
Instant psychological well-being on the OSCE day	
Stress level, Mdn [IQR]	52 [31, 70]
Emotional valence level, Mdn [IQR]	49 [34, 59]
Self-confidence level, Mdn [IQR]	45 [26, 56]
Instant psychological well-being, M (± SD)	46.2 (± 17.1)
Performance at the OSCE, M (± SD)	21.8 (± 4.0)

Data are presented as mean with standard deviation (M ± (SD)), median with interquartile (Mdn [IQR]), or number of observations and percentage (n (%)). Coping behavior scores were extracted from the brief cope inventory, with scores ranging from 1 to 4. Data for the global physical activity questionnaire and Pittsburgh sleep quality index were analyzed for 453 (94% of the cohort) and 477 (99%) students, respectively (due to aberrant responses). Instant psychological well-being indicators were evaluated through visual analogue scales, which results translate in a score from 0 to 100. Performance was assessed by independent university examiners, performance score may range from 0 to 40.

*MET* metabolic equivalent of task.

### Instant psychological well-being (from 0 to 100)

Instant psychological well-being was positively associated with male gender (compared to female, β = 7.44, 95% CI [4.34, 10.54], *p* < 0.001), with the level of positive thinking (β = 6.69, 95% CI [4.52, 8.87], *p* < 0.001), and with the level of physical activity (β = 0.001, 95% CI [0.00, 0.00], *p* = 0.027) (Table 2).

**Table 2:** Multivariable regression model to identify the association between each coping and health-related behavior and instant psychological well-being (from 0 to 100).

	β	95% CI [,]	*p* value	Effect size with 95% CI
Characteristics				
Gender (male)	7.440	[4.34, 10.54]	**< 0.001**	**0.44 [0.26, 0.62]**
BMI	− 0.314	[− 0.78, 0.15]	0.188	− 0.05 [− 0.14, 0.03]
Age	− 0.782	[− 1.51, − 0.05]	**0.036**	**− 0.08 [− 0.16, − 0.01]**
Coping behaviors				
Active resolution	0.899	[− 1.01, 2.81]	0.355	0.04 [− 0.04, 0.12]
Positive thinking	6.693	[4.52, 8.87]	**< 0.001**	**0.25 [0.17, 0.33]**
Social support	− 0.604	[− 3.25, 2.04]	0.653	− 0.02 [− 0.11, 0.07]
Avoidance	− 9.331	[− 12.90, − 5.76]	**< 0.001**	**− 0.23 [− 0.32, − 0.14]**
Health− related behaviors				
Physical activity	0.001	[0.00, 0.00]	**0.027**	**0.09 [0.01, 0.17]**
Sleep disturbance	− 1.082	[− 1.53, − 0.63]	**< 0.001**	**− 0.19 [− 0.27, − 0.11]**

Significant relationships of the model analyses are in bold, *p* value of the model: < 0.002, adjusted R^2^ was 0.336 (number of complete observations = 437).

*BMI* body mass index.

Instant psychological well-being was negatively associated with age (β = − 0.78, 95% CI [− 1.51, − 0.05], *p* = 0.036), with the level of avoidance (β = − 9.33, 95% CI [− 12.90, − 5.78], *p* < 0.001), and with the level of sleep disturbance (β = − 1.08, 95% CI [− 1.52, − 0.63], *p* < 0.001) (Table 2).

### Performance (from 0 to 40)

OSCE performance was negatively associated with the level of avoidance (β = − 1.37, 95% CI [− 2.39, − 0.35], *p* < 0.01; Table 3).

**Table 3:** Multivariate regression model to identify the association between each coping and health-related behavior and performance (from 0 to 40).

	β	95% CI [,]	*p* value	Effect size with 95% CI
Characteristics				
Gender (male)	− 0.885	[− 1.77, 0.00]	0.050	− 0.22 [− 0.44, 0.00]
BMI	− 0.053	[− 0.19, 0.08]	0.432	− 0.04 [− 0.14, 0.06]
Age	− 0.135	[− 0.34, 0.07]	0.206	− 0.06 [− 0.16, 0.03]
Coping behaviors				
Active resolution	0.023	[− 0.52, 0.57]	0.935	4.15e^−03^ [− 0.10, 0.10]
Positive thinking	0.241	[− 0.38, 0.86]	0.446	0.04 [− 0.06, 0.13]
Social support	0.064	[− 0.82, 0.70]	0.867	− 9.45e^−03^ [− 0.12, 0.10]
Avoidance	− 1.369	[− 2.39, − 0.35]	**0.009**	**− 0.14 [− 0.25, − 0.04]**
Health-related behaviors				
Physical activity	− 0.0001	[− 0.00, 0.00]	0.167	− 0.07 [− 0.17, 0.03]
Sleep disturbance	− 0.106	[− 0.23, 0.02]	0.107	− 0.08 [− 0.17, 0.02]

Significant relationships of the model analyses are in bold, *p* value of the model: < 0.002, adjusted R^2^ was 0.040 (number of complete observations = 437).

*BMI* body mass index.

### Stress, emotional valence, self-confidence correlations

Stress level was negatively correlated with positive thinking and physical activity, and positively correlated with social support, avoidance, and sleep disturbance (Table 4). The emotional valence level was positively correlated with positive thinking and active resolution, and negatively correlated with avoidance and sleep disturbance. The level of self-confidence was positively correlated with positive thinking, active resolution, and physical activity, and negatively correlated with social support, avoidance, and sleep disturbance (Table 4; Appendices 6 and 7).

**Table 4:** Spearman correlations exploring associations between coping behaviors (positive thinking, active resolution, social support, avoidance) and health-related behaviors with instant psychological well-being and with performance.

	VAS Stress		VAS valence		VAS self-confidence		OSCE performance	
	R	*p* value	R	*p* value	R	*p* value	R	*p* value
Coping behaviors								
Active resolution	0.02	0.72	**0.13**	** < 0.01**	**0.09**	**0.04**	-0.04	0.45
Positive thinking	**− 0.25**	** < 0.001**	**0.36**	** < 0.001**	**0.30**	** < 0.001**	0.03	0.57
Social support	**0.25**	** < 0.001**	-0.09	0.05	**− 0.12**	**0.01**	-0.03	0.56
Avoidance	**0.26**	** < 0.001**	**− 0.29**	** < 0.001**	**− 0.31**	** < 0.001**	**− 0.11**	**0.02**
Health-related behaviors								
Physical activity	**− 0.21**	** < 0.001**	0.09	0.06	**0.15**	** < 0.01**	-0.09	0.058
Sleep disturbance	**0.21**	** < 0.001**	**− 0.28**	** < 0.001**	**− 0.29**	** < 0.001**	**− 0.11**	**0.01**

Coping behavior scores were extracted from the brief cope inventory. Instant psychological well-being was assessed through a multi-dimensional visual analogue scale of 100 mm (stress, emotional valence, self-confidence). Emotional valence characterized the feeling associated with the stress (from 0: “the most negative feeling possible” to 100: “the most positive feeling possible”). The Pittsburgh sleep quality index questionnaire was used to assess sleep disturbance, a higher score corresponding to poorer sleep. The global physical activity questionnaire identified the level of physical activity in MET-minutes/week. R: correlation factor.

Significant values are in [bold].

## Discussion

This exploratory study aimed to assess how coping and health-related behaviors executed by medical students prior to the OSCE were associated with their psychological well-being and performance on the examination day. The results emphasize the significance of taking both of these behaviors into account when considering well-being and performance in a real-life stressful context for undergraduate medical students.

In regards to coping behaviors, the present study found that positive thinking was positively associated with a substantial 7% increase in well-being (ranging from 4.5 to 8.9%) on the day of the OSCE. Elevated well-being just before the OSCE should help students to mobilize their inner resources. Detailed correlations revealed that positive thinking correlated with low stress level, a stress perceived more positively, and a high level of self-confidence. Similarly, active resolution correlated positively with a stress perceived more positively, and self-confidence. In a literature review focusing on medical students, Dyrbye et al. stated that positive reinterpretation (a part of positive thinking) and problem-solving (a part of active resolution) lead to adaptation, reduce anxiety, and have long-term positive influence on mental health^41^. Similarly, the present findings, support the value of offering preventive programs based on positive thinking and active resolution to medical students for increased instant psychological well-being in the context of a stressful exam. High level of well-being might help to foster clear thinking, focus, and memory recall. Encouraging well-being before exams may further reinforces self-care and positively impacts student’s overall well-being and life satisfaction. Positive thinking may be promoted by implementing preventive programs during medical curriculum that have already shown promising results in terms of stress reduction in students and healthcare providers^42,43^. Active resolution may be promoted by implementing time management training, which has by itself proven its efficacy in terms of lowering students’ stress levels^44^. Additional randomized controlled trials might be undertaken to assess the efficacy of preventive programs in improving psychological well-being within the context of examinations and academic achievement.

Conversely, the present results showed that engaging in avoidance was associated with a substantial − 9% decrease in well-being (ranging from − 13 to − 6%). Detailed correlations revealed that avoidance correlated with high stress level, a stress perceived more negatively, and a low level of self-confidence. Previous findings reported that behavioral disengagement, which is part of avoidance coping behaviors, causes a high stress level and is associated with depressive symptoms in the general life of college students^3,45,46^. While lowering avoidance behaviors seems difficult in practice, informing students about these findings (e.g., through lectures, posters, social networks) is an important step to help students identify their own maladaptive behaviors (i.e. self-distraction, self-blame, denial, substance use, and behavioral disengagement). Subsequently, offering specific guidance could help them shift from avoidance to positive thinking and active resolution.

In regards to health-related behaviors, the results of the present study showed that physical activity was associated with higher instant psychological well-being on the OSCE day. More specifically, the level of physical activity correlated with a low level of stress and a high level of self-confidence. This confirms previous studies that reported that physical activity reduces stress level and boosts self-confidence in the day-to-day life of medical students^19,20,47^. The present study also found that sleep disturbance was associated with a lower instant psychological well-being. More specifically, the level of sleep disturbance correlated with a higher stress level, a stress perceived more negatively, and lower self-confidence. These findings are consistent with previous findings on the relationships between sleep and stress in medical students in their daily life^16,17^. Sleep deprivation is associated with chronic and serious health problems such as cardiovascular diseases^48–50^, and more than half of the students herein reported sleep disturbances, suggesting that they could also be at high risk for health issues. In addition, students adopted sedentary behaviors for more than 10 h per day, which are known to be detrimental for long-term physical health^51,52^. Programs promoting good health hygiene have shown promising results^53,54^. For example, White et al. offered to public health students a self-care intervention available online focusing on nutrition, physical activity, mental health, and social support; this led to an improvement in health behaviors over the course of the semester^54^. Further randomized controlled trials should be conducted to evaluate the efficacy of health programs in enhancing psychological well-being notably in specific real-life stressful situations such as examination settings.

The secondary aim of the present study was to determine how OSCE performance was associated with coping and health-related behaviors. It was found that OSCE performance was negatively associated with avoidance, a result in line with studies focusing on the effects of coping styles on academic performance^10,11,25^. Engaging in avoidance coping behavior was, on average, associated with a substantial − 1.4 points decrease in performance, equivalent to a − 3.5% (from − 1 to − 6%) reduction in OSCE performance. Considering the substantial weight of this major assessment in students' ranking and its impact on medical specialty selection for fellowships, such variation may have profound implications in student’s professional life. As mentioned earlier, educating students about the negative impacts of avoidance on both well-being and academic performance can be an initial step in aiding them to recognize their counterproductive behaviors. There was also a significant negative correlation between sleep disturbance and OSCE performance. Previous studies found that sleep disturbance seems to be detrimental to general academic performance^55,56^. For instance, a trial focusing on 20 anesthesia residents in a simulated crisis scenario, found that sleep-loss was responsible for impaired non-technical skills and decreased self-confidence^56^; herein a negative correlation between sleep disturbance and self-confidence was found, confirming this result in a larger and younger cohort of medical students.

Finally, there was no association between physical activity and performance. Previous studies have investigated the association between physical activity and academic performance on secondary school-aged students^57–59^. Franz et al. found no association between performance and level of physical activity in secondary school students^59^; conversely, a study focusing on medical and health sciences students found that the probability of having a good grade was higher among the students who met the minimum level of physical activity^57^. We recommend conducting additional research by incorporating objective physical activity measurements, in addition to using questionnaires, as such an approach would provide a better understanding of these potential relationships within examination settings.

The present study has some limitations. Firstly, the questionnaires on physical activity, sleep quality, and coping behaviors assessed self-reported behaviors during a dissimilar temporal window (2 weeks before the examination, 1 month before the examination, or general level, respectively). This was done in order to respect the instructions of the validated questionnaires; the temporal aspect of the questionnaires could have been harmonized but this may have brought into doubt the validity of the results. Secondly, this was a single-center study, these results could benefit from an external validation in another cohort and/or another examination context. Thirdly, low-adjusted R^2^ were reported. While these values are similar to those reported in behavioral and psychology research, this means that the percentage of variance explained may be low. A more comprehensive understanding could thus be carried out by taking into account variables related to mental health (such as anxiety trait, or the presence of depressive symptoms) and/or revision techniques put in place by students. Fourthly, it cannot be completely ruled out that answering a questionnaire just before an exam could influence the emotional state and increase cognitive load, affecting students' abilities to perform their OSCE.

This study also has several strengths. Firstly, it is the first to investigate the relationships between psychological well-being, performance, coping, and health-related behaviors in a single protocol. Secondly, the inclusion of over 480 students enables the exploration of these relationships in a relatively large sample. Thirdly, nearly all students participated (only one refusal), ensuring a high level of internal validity and minimizing selection bias.

As a final remark, while our study did not allow us to draw conclusions on causal relationships, some interpretations might be inferred from the observed associations. From a temporal aspect, it appears more likely that coping behaviors and health-related behaviors adopted weeks before the exam influence subsequent levels of well-being and performance on the OSCE day. Nonetheless, there is still the possibility that a student who generally performs poorly academically may experience lower well-being and resort to avoidance behavior to evade upcoming situations where they anticipate failure. Future studies delineating causal and potential bi-directional influences should be addressed to identify the best way to assist medical students.

To conclude, this study reports how medical students’ coping and health-related behaviors during the weeks preceding the OSCE are associated with their psychological well-being on the day of the examination. The main findings were that the level of instant psychological well-being was positively associated with positive thinking and physical activity, while it was negatively associated with avoidance and sleep disturbance. In addition, OSCE performance was negatively associated with avoidance. These findings emphasize the importance of promoting physical activity and good sleep hygiene at universities, supporting the development of stress management programs that focus on positive thinking for medical students. They also highlight the significance of identifying and assisting students who engage in avoidance coping behaviors.

### Supplementary Information

Supplementary Information.

## Data Availability

The datasets used and/or analyzed during the current study are available from the corresponding author on reasonable request.

## Abbreviations

BCI: Brief cope inventory
BMI: Body mass index
GPAQ: Global physical activity questionnaire
MET: Metabolic equivalent of task
OSCE: Objective structured clinical examination
PSQI: Pittsburgh sleep quality index
VAS: Visual analogue scale
HO: World Health Organization
