# Supplementary material for: Associations of coping and health-related behaviors with medical students’ well-being and performance during objective structured clinical examination
Source: Sci Rep. 2024 May 17;14:11298. doi: 10.1038/s41598-024-61800-1 (PMC11101410; doi:10.1038/s41598-024-61800-1)
Supplement: Supplementary file 1 — Supplementary Information. [file 41598_2024_61800_MOESM1_ESM.docx]

# **Optional supplementary information files**

**Appendix 1. OSCE supplementary information**

- 1. **OSCE organization**

The OSCE was organized in circular circuits of five consecutive examination scenarios. Each scenario included a standardized patient, and had to be completed within seven minutes. Immediately after, the examiner provided a two-minute feedback using a cognitive aid. There were four identical circuits to simultaneously admit a wave of 20 students. The topics of stations covered specific medical fields taught during the fourth year of medical studies. One examiner per station rated the student’s performance following a scenario-specific pre-determined grid of binary items (16-30 items per station). Each grid had a maximum score of 40 points.

- 1. **List of scenario themes employed**

| **Types** | **Themes** |
| --- | --- |
| 1 - History taking | Psoriasis  Pancytopenia  Tuberculosis |
| 2 - Clinical examination | Scapular pain  Shortness of breath  Hypertension |
| 3 - Comprehensive physical exams | Hypercalcemia  Providing information before bronchial fiberoptic  Thrombocytosis |
| 4 - Announcement | Medical advice for travel  Type 2 diabetes  Need for a CT scan to explore the pancreas |
| 5 - Technical procedure | Performing otoscopy  Performing stiches  Inserting peripheral intra-venous line |

**Appendix 2. Visual Analogues Scales (VAS)**

Students were asked to answer three questions using 100mm visual analogue scales (VAS) by moving the cursor in the corresponding direction of their feelings. In the following illustrations the cursors are positioned at their initial position. In order to help understanding how to report their answer, students received the following instruction, for the first VAS instruction: “For the following questions, please report your answers by moving the cursor.” The cursor was set to "Zero" for the VAS stress and VAS self-confidence. The cursor was set to 50 for the VAS emotional valence corresponding to a neutral position.

**VAS stress**

What is your stress level at this moment?

*Extreme Maximum*

Zero

**VAS emotional valence**

What is the emotional valence associated with your stress at this moment?

The most positive feeling possible

The most negative feeling possible

**VAS self-confidence**

What is your current level of self-confidence?

Zero

Maximum

**Appendix 3.** **Models’ assumptions**

- 1. **Data distribution**

A.1. Instant well-being

**
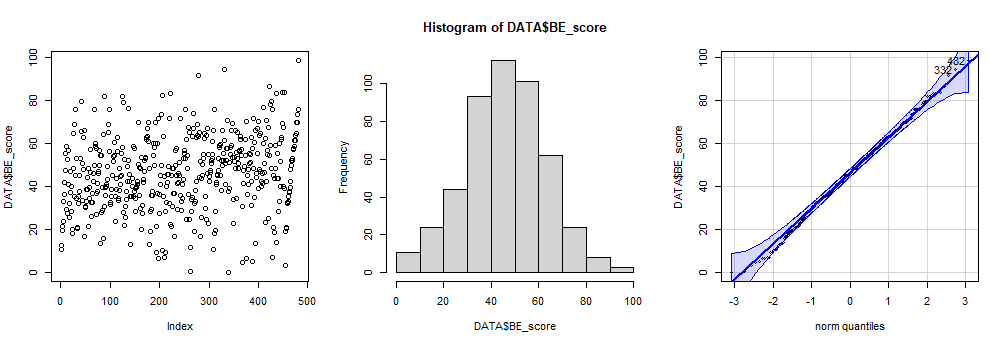
**

A.2. OSCE performance

**
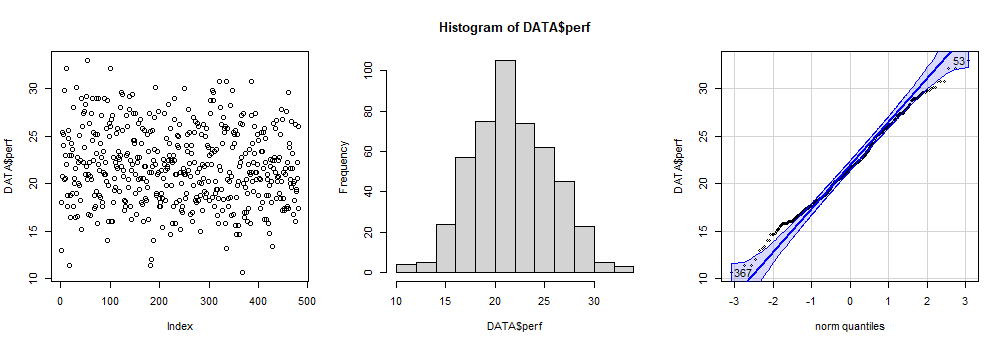
**

- 1. **Linear regression assumptions**

B.1. Visual diagnostic of the instant well-being model

**
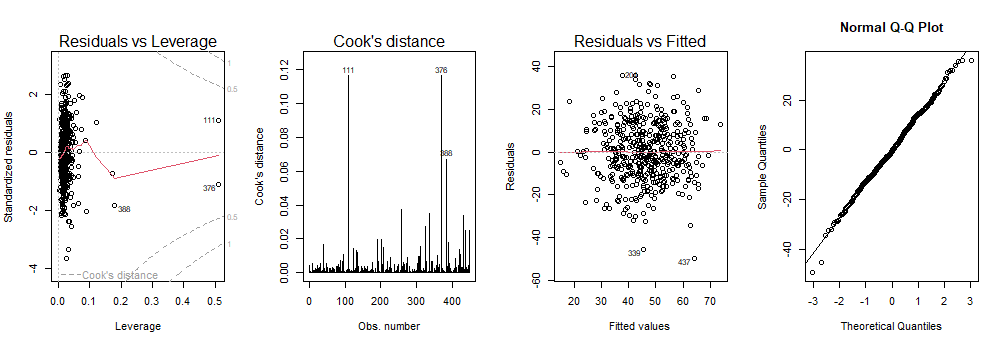
**

B.2. Visual diagnostic of the performance mode**
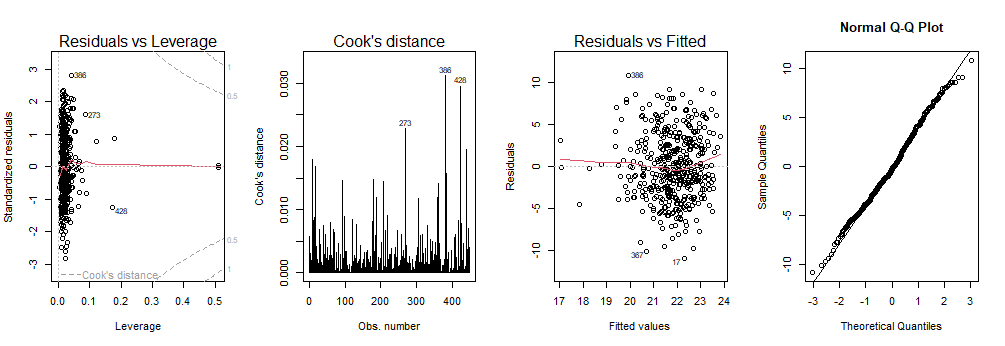
**

**Appendix 4. Pittsburgh Sleep Quality Index (PSQI) results**

|  | **Mean (± SD)** | **Range** |
| --- | --- | --- |
| During the past month, when have you usually gone to bed at night? | 23.5 (± 0.9) = 11:30 PM | 8:30 PM - 4:00 AM |
| During the past month, how long (in minutes) did it usually take you to fall asleep? | 21.3 (± 21.3) min | 0 - 180min |
| During the past month, when did usually you get up in the morning? | 7.34 (± 0.98) = 7:20 AM | 5:00 AM - 5:00 PM |
| During the past month, how many hours of actual sleep did you get at night? | 7.20 (± 0.95) = 7h15 | 4 - 10h |
| During the past month, how often have you had trouble sleeping because you...  Following questions could be answered by:  1. Not during the past month  2. Less than once a week  3. Once or twice a week  4. Three or more times a week |  | |
| Could not get to sleep within 30 minutes, n (%) | 1. 152 (32%) | |
|  | 1. 135 (28%) | |
|  | 1. 88 (18%) | |
|  | 1. 102 (21%) | |
| Woke up in the middle of the night or early morning, n (%) | 1. 125 (26%) | |
|  | 1. 113 (24%) | |
|  | 1. 131 (27%) | |
|  | 1. 108 (23%) | |
| Had to get up to use the bathroom, n (%) | 1. 275 (58%) | |
|  | 1. 90 (19%) | |
|  | 1. 64 (13%) | |
|  | 1. 48 (10%) | |
| Could not breathe comfortably, n (%) | 1. 423 (88%) | |
|  | 1. 28 (6%) | |
|  | 1. 16 (3%) | |
|  | 1. 10 (2%) | |
| Coughed or snored loudly, n (%) | 1. 411 (86%) | |
|  | 1. 35 (7%) | |
|  | 1. 19 (4%) | |
|  | 1. 12 (3%) | |
| Felt too cold, n (%) | 1. 399 (84%) | |
|  | 1. 57 (12%) | |
|  | 1. 19 (4%) | |
|  | 1. 2 (0.4%) | |
| Felt too hot, n (%) | 1. 107 (22%) | |
|  | 1. 122 (26%) | |
|  | 1. 172 (36%) | |
|  | 1. 76 (16%) | |
| Experienced bad dreams, n (%) | 1. 306 (64%) | |
|  | 1. 97 (20%) | |
|  | 1. 49 (10%) | |
|  | 1. 25 (5%) | |
| Had pain, n (%) | 1. 413 (86%) | |
|  | 1. 44 (9%) | |
|  | 1. 11 (2%) | |
|  | 1. 9 (2%) | |
| Reported other reasons: Open and optional question, 259 answers. The five most recurring words:   1. Stress (n=64) 2. Thoughts (n=38) 3. Revisions (n=31) 4. Noise (n=21) 5. Problems (n=14)   n (%) | 1. 2 (0.7%) | |
|  | 1. 51 (19%) | |
|  | 1. 98 (38%) | |
|  | 1. 108 (42%) | |
| During the past month, how would you rate your sleep quality overall?  (From 1 = Very good to 4 = Very bad), n (%) | 1. 47 (10%) | |
|  | 1. 168 (35%) | |
|  | 1. 202 (42%) | |
|  | 1. 60 (12%) | |
| Following questions could be answered by:  1. Not during the past month  2. Less than once a week  3. Once or twice a week  4. Three or more times a week |  | |
| During the past month, how often have you taken medicine (prescribed or over-the-counter) to help you sleep?  n (%) | 1. 424 (89%) | |
|  | 1. 22 (4%) | |
|  | 1. 13 (3%) | |
|  | 1. 19 (4%) | |
| During the past month, how often have you had trouble staying awake while driving, eating meals, or engaging in social activities?  n (%) | 1. 284 (60%) | |
|  | 1. 100 (21%) | |
|  | 1. 66 (14%) | |
|  | 1. 27 (5%) | |
| During the past month, how much of a problem has it been for you to keep up enthusiasm to get things done?  n (%) | 1. 95 (20%) | |
|  | 1. 230 (48%) | |
|  | 1. 110 (23%) | |
|  | 1. 42 (9%) | |

Pittsburgh Sleep Quality Index (PSQI) results. The PSQI score ranged from 0 to 21, a high score translating into a high level of sleep disturbance. A score ≥ 6/21 was the threshold to screen for sleep disturbance. Five questionnaires were not included in the final analysis, due to aberrant answers, e.g., answer for sleeping hours equal to “0”. In the open question asking about reasons for sleep trouble, the most occurring words (excluding linking words, e.g., “the”, “of”) are presented as follows: English translation (number of occurrences). Numbers of occurrences were identified using the R software.

**Appendix 5. Global Physical Activity Questionnaire (GPAQ) results**

| **Question** | **Number of Yes (%)** | **Focused question** | **Mean (± SD)** | **Range** |
| --- | --- | --- | --- | --- |
| Does your work involve vigorous-intensity activity that causes large increases in breathing or heart rate like [carrying or lifting heavy loads, digging or construction work] for at least 10 minutes continuously? | 26 (5.7) | In a typical week, on how many days do you do vigorous-intensity activities as part of your work? | 2.9 (± 1.9) | 1 - 6 |
|  |  |  |  |  |
|  |  | How much time (minutes) do you spend doing vigorous-intensity activities at work on a typical day? | 78.3 (± 57.3) | 15 - 270 |
| Does your work involve moderate-intensity activity that causes small increases in breathing or heart rate such as brisk walking *[or carrying light loads*] for at least 10 minutes continuously? | 134 (29.5) | In a typical week, on how many days do you do moderate-intensity activities as part of your work? | 3.7 (± 1.7) | 1 - 7 |
|  |  | How much time (minutes) do you spend doing moderate-intensity activities at work on a typical day? | 77.1 (± 57.1) | 10 - 270 |
| Do you walk or use a bicycle (*pedal cycle*) for at least 10 minutes continuously to get to and from places? | 409 (90.0) | In a typical week, on how many days do you walk or bicycle for at least 10 minutes continuously to get to and from places? | 5.5 (± 1.5) | 1 - 7 |
|  |  | How much time (minutes) do you spend walking or bicycling for travel on a typical day? | 42.3 (± 28.0) | 5 - 240 |
| Do you do any vigorous-intensity sports, fitness or recreational (*leisure*) activities that cause large increases in breathing or heart rate like [*running or football,]* for at least 10 minutes continuously? | 355 (78.3) | In a typical week, on how many days do you do vigorous-intensity sports, fitness or recreational (*leisure*) activities? | 2.7 (± 1.5) | 1 - 7 |
|  |  |  |  |  |
|  |  | How much time (minutes) do you spend doing vigorous-intensity sports, fitness or recreational activities on a typical day? | 64.8 (± 38.1) | 10 - 360 |
| Do you do any moderate-intensity sports, fitness or recreational *(leisure*) activities that causes a small increase in breathing or heart rate such as brisk walking (*cycling, swimming, volleyball*) for at least 10 minutes continuously? | 293 (64.7) | In a typical week, on how many days do you do moderate-intensity sports, fitness or recreational (*leisure*) activities? | 2.8 (± 1.8) | 1 - 7 |
|  |  |  |  |  |
|  |  | How much time (minutes) do you spend doing moderate-intensity sports, fitness or recreational (*leisure*) activities on a typical day? | 54.6 (± 37.1) | 10 - 300 |
| How much time (hours) do you usually spend sitting or reclining on a typical day? | | | 9.0 (± 2.4) | 0 - 16 |
| **GPAQ total score (MET-minutes/week)** | | | 2818 (± 2083) | 0 - 12960 |
| **minutes-weeks** | | | 554 (± 403) | 0 - 2280 |

Global Physical Activity Questionnaire (GPAQ) results. From these data, a score in MET-minute/week can be calculated. The WHO recommends a minimal physical activity of 600 MET-minutes per week, which correspond to 150 minutes of moderate-intensity physical activity per week, or an equivalent. Twenty-nine questionnaires were excluded from further analysis due to missing data and/or aberrant answers (e.g., a subdomain of physical activity greater than 16 hours per day).

**Appendix 6.** **Correlations between coping behaviors and instant psychological well-being.**

**A. Positive thinking**


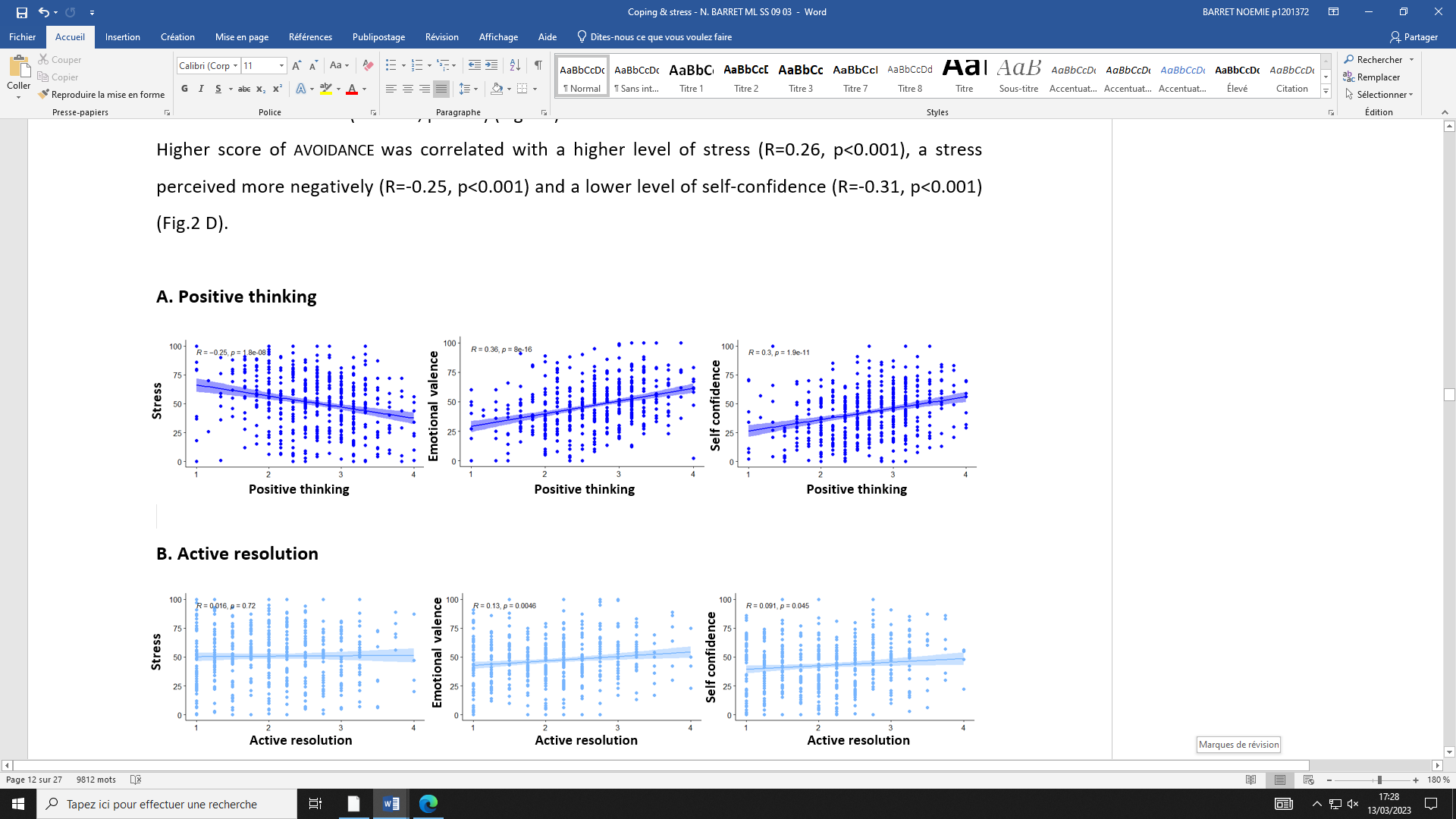


**B. Active resolution**


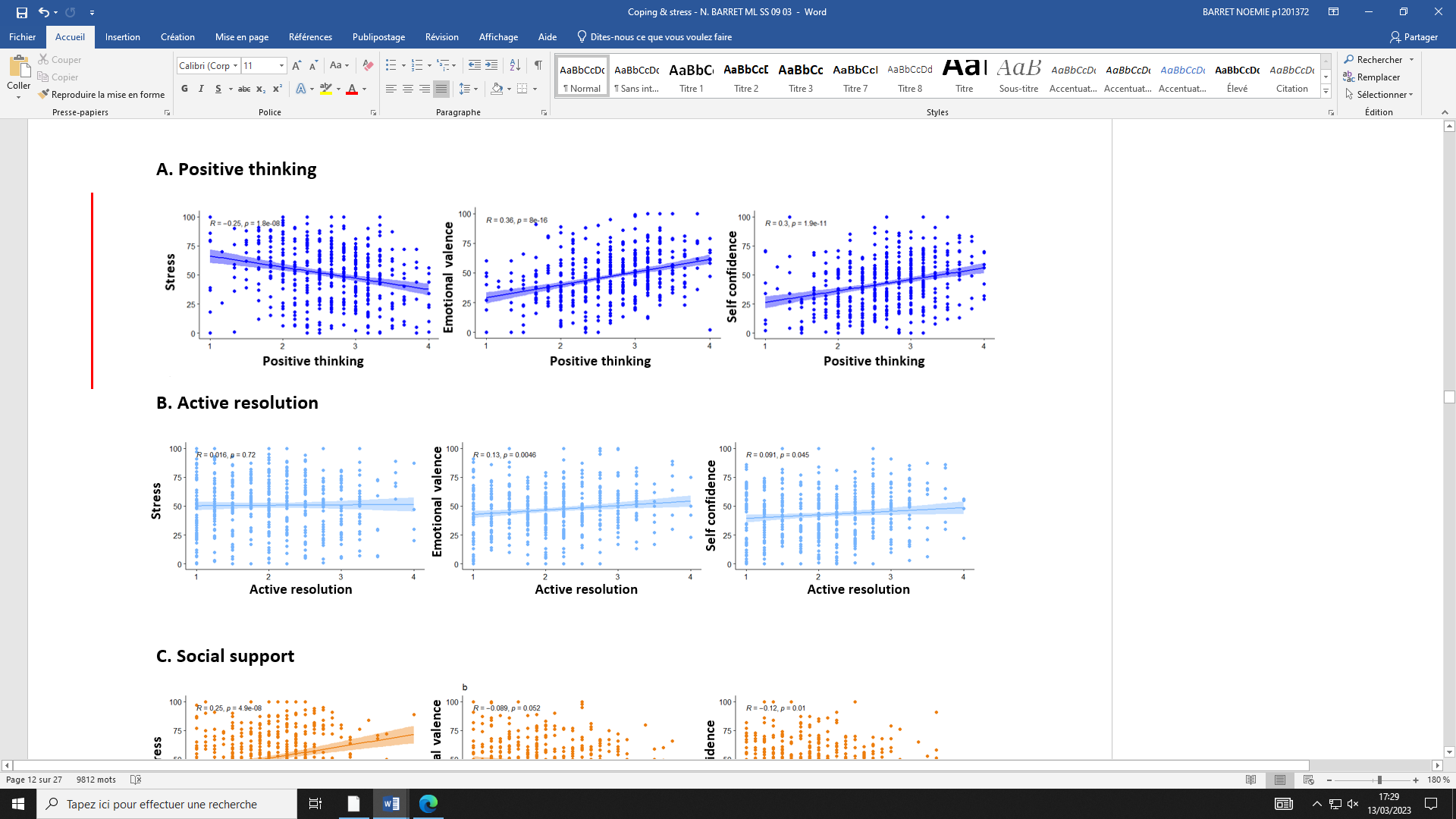


**C. Social support**


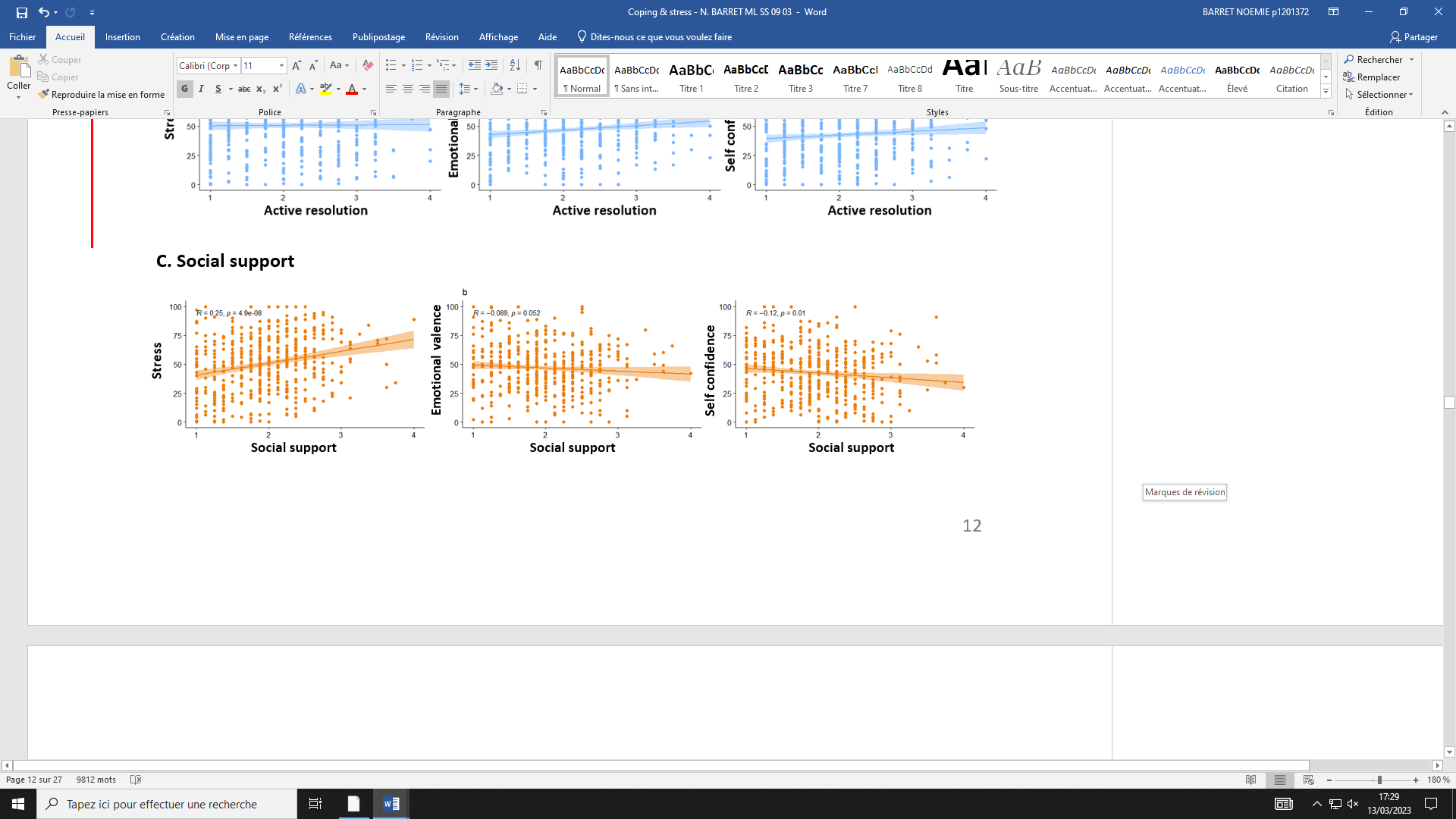


**D. Avoidance**


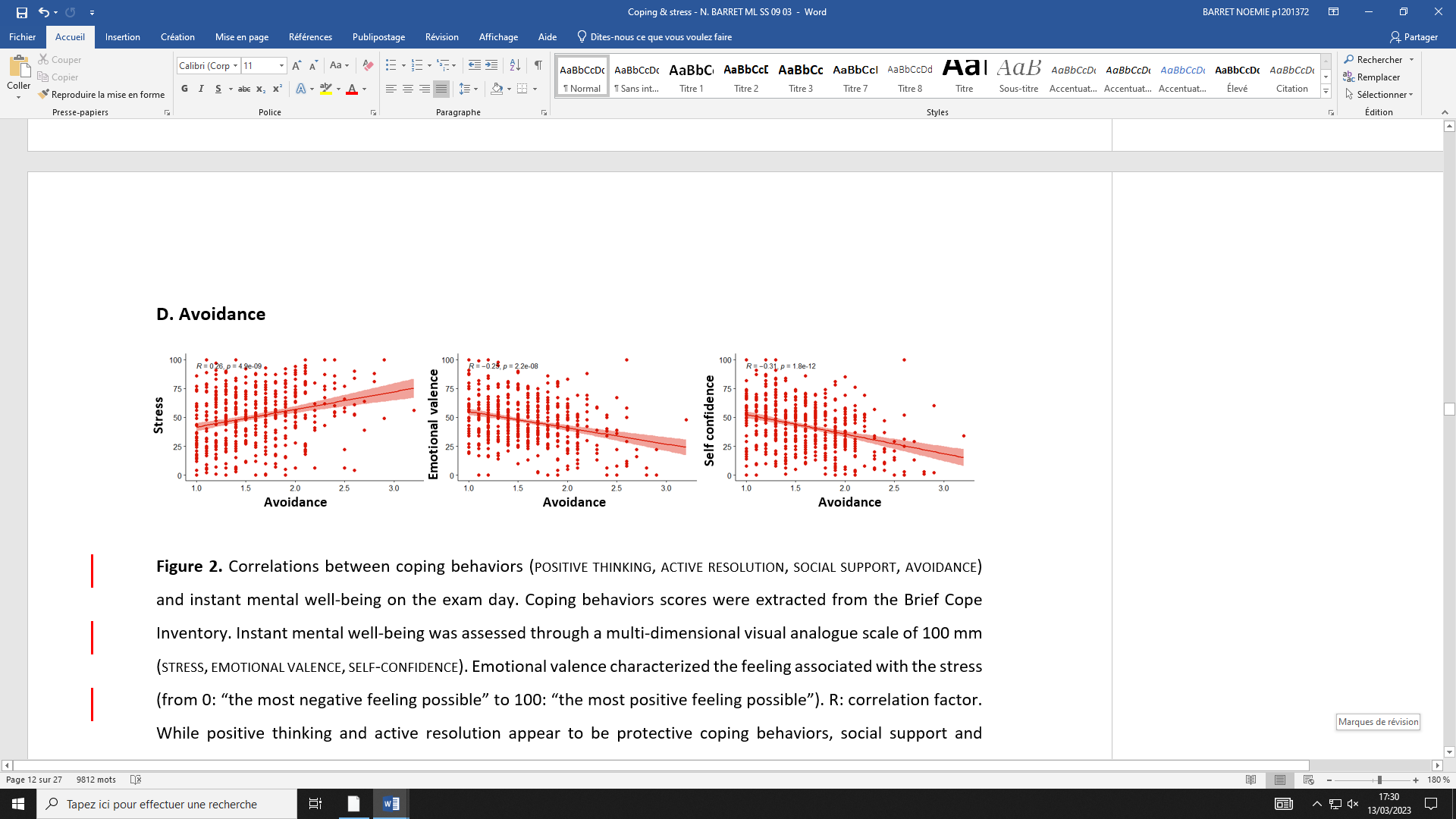


Spearman correlations between coping behaviors (positive thinking, active resolution, social support, avoidance) and instant psychological well-being on the examination day. Coping behavior scores were extracted from the Brief Cope Inventory. Each component of the instant psychological well-being was assessed through a multi-dimensional visual analogue scale of 100 mm, giving a score ranging from 0 to 100 (stress: 0 “no stress” to 100 “very high” level of stress; emotional valence: 0 “very negative” to 100 “very positive” feeling; self-confidence: 0 “very low” to 100 “very high” self-confidence). Engagement in each behavior ranges from 1 to 4. R: correlation factor.

**Appendix 7. Correlations between health-related behaviors and the instant psychological well-being.**

**A. Sleep disturbance**


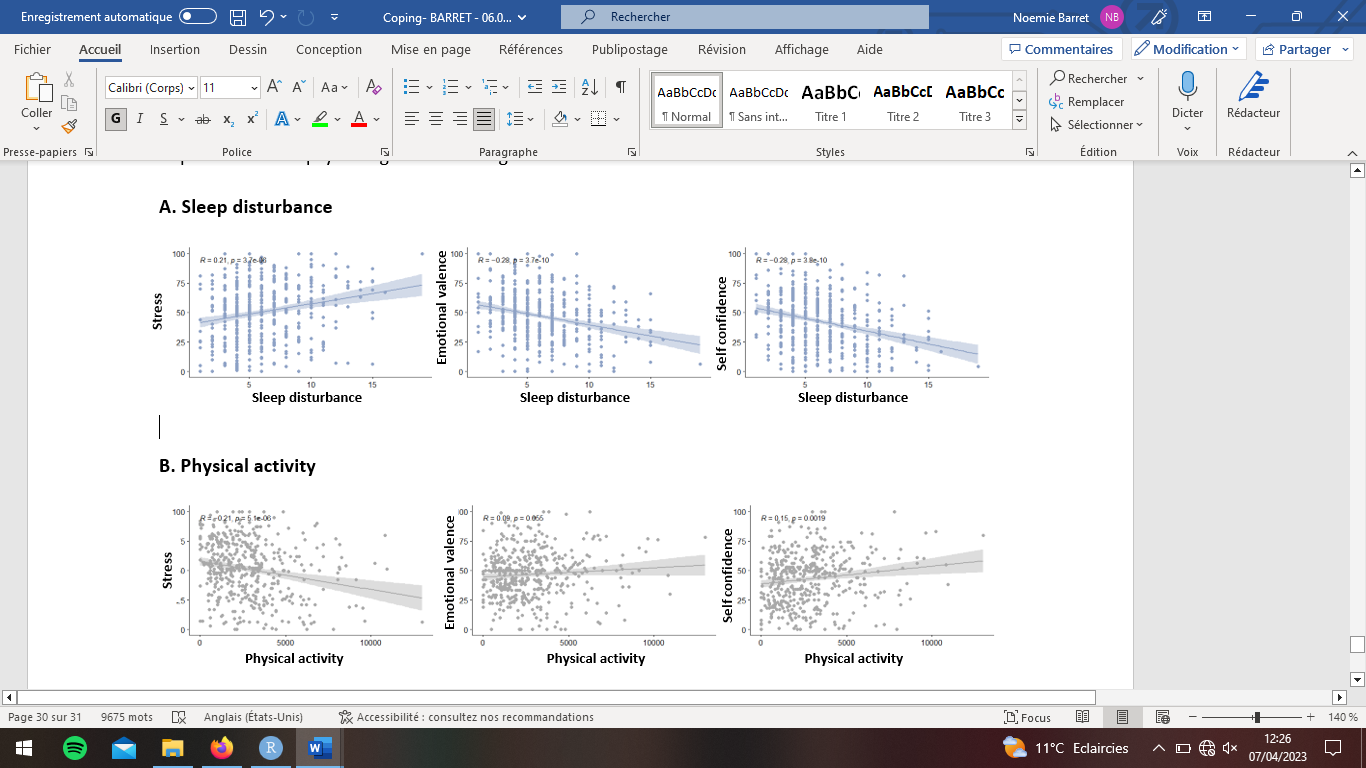


**B. Physical activity**


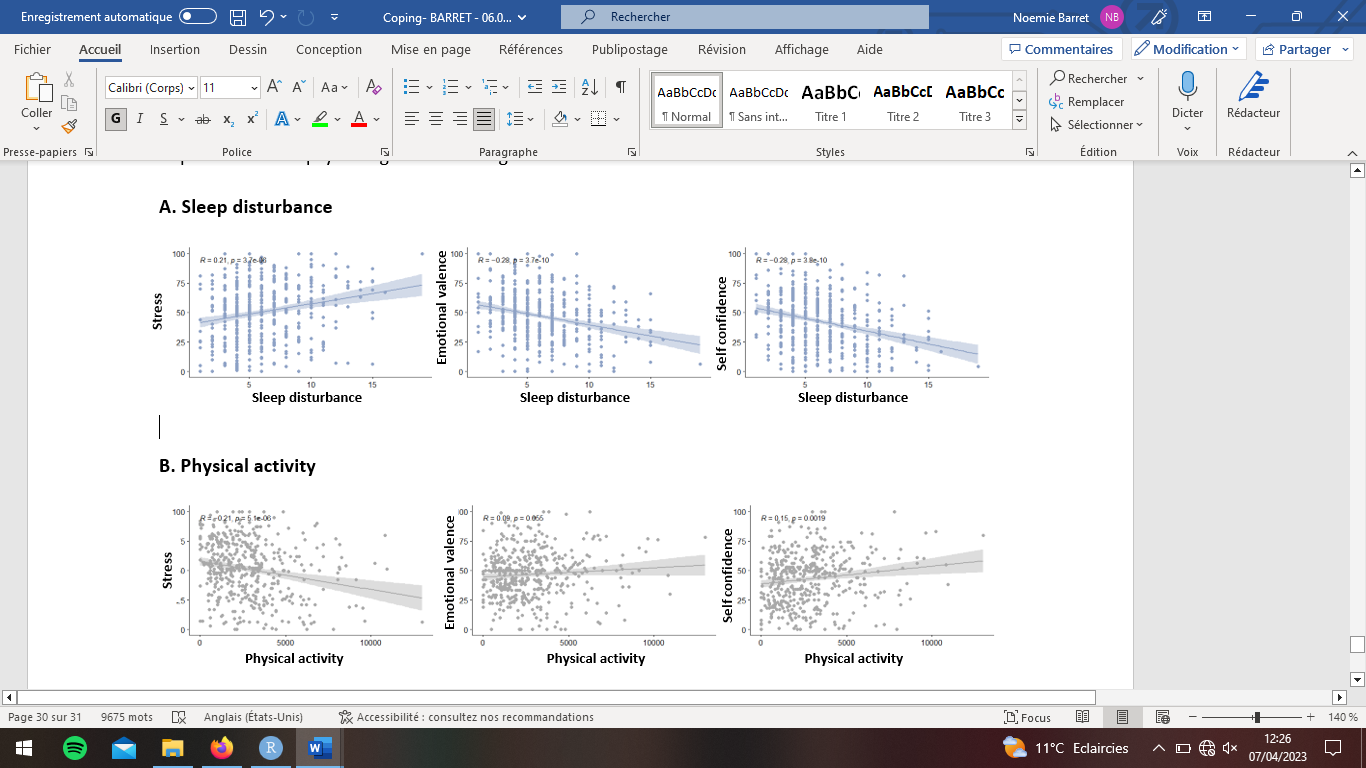


Spearman correlations between health-related behaviors (sleep disturbance, physical activity) and the instant psychological well-being on the examination day. Each component of the instant psychological well-being was assessed through a multi-dimensional visual analogue scale of 100 mm, giving a score ranging from 0 to 100 (stress: 0 “no stress” to 100 “very high” level of stress; emotional valence: 0 “very negative” to 100 “very positive” feeling; self-confidence: 0 “very low” to 100 “very high” self-confidence). The PSQI questionnaire was used to assess sleep disturbance, a higher score corresponding to poorer sleep. The GPAQ questionnaire identified the level of physical activity in MET-minutes/week, a higher score corresponding to a higher level of physical activity. R: correlation factor.
